# Comparison of optical coherence tomography angiography and narrow-band imaging using a bimodal endoscope

**DOI:** 10.1117/1.JBO.25.3.032003

**Published:** 2019-09-27

**Authors:** Lara M. Wurster, Simon Kretschmer, Jan Jäger, Fabian Placzek, Laurin Ginner, Wolfgang Drexler, Çağlar Ataman, Rainer A. Leitgeb, Hans Zappe

**Affiliations:** a Medical University of Vienna, Center for Medical Physics and Biomedical Engineering, Vienna, Austria; b Medical University of Vienna, Christian Doppler Laboratory for Innovative Optical Imaging and Its Translation to Medicine, Vienna, Austria; c University of Freiburg, Gisela and Erwin Sick Chair of Micro-optics, Department of Microsystems Engineering, Freiburg, Germany

**Keywords:** optical coherence tomography, endoscopy, angiography, narrow-band imaging

## Abstract

We present coregistered images of tissue vasculature that allow a direct comparison between the performance of narrow-band imaging (NBI) and optical coherence tomography angiography (OCTA). Images were generated with a bimodal endomicroscope having a size of $15\times 2.4\times 3.3\;{\mathrm{mm}}^{3}$ (l,w,h) that combines two imaging channels. The white light imaging channel was used to perform NBI, the current gold standard for endoscopic visualization of vessels. The second channel allowed the simultaneous acquisition of optical coherence tomography (OCT) and OCTA images, enabling a three-dimensional (3-D) visualization of morphological as well as functional tissue information. In order to obtain 3-D OCT images scanning of the light-transmitting fiber was implemented by a small piezoelectric tube. A field of view of ∼1.1  mm was achieved for both modalities. Under the assumption that OCTA can address current limitations of NBI, their performance was studied and compared during *in vivo* experiments. The preliminary results show the potential of OCT regarding an improved visualization and localization of vessel beds, which can be beneficial for diagnosis of pathological conditions.

## 1 Introduction

The current gold standard for endoscopic visualization of vessel networks is narrow-band imaging (NBI), which requires the integration of a filter in a standard commercially available white-light endoscope. The method was developed in 1999 and first described in 2001 for gastrointestinal applications.^1^ The usage of a filter narrows down the bandwidth of the transmitted white light to a certain wavelength region.^2^ Most commonly, wavelengths around 415 nm (blue) or 540 nm (green) are chosen since these wavelengths correspond to the hemoglobin absorption band.^3^ Consequently, light is absorbed by the blood running through the vessels and the contrast between the mucosal layer and vasculature is enhanced. Choosing a longer wavelength will lead to an increased penetration depth inside the tissue and therefore enables visualization of slightly deeper located vessels. A change from 415 to 540 nm, for example, has an increased penetration depth of 70  μm (240  μm, compared to 170  μm).^2^ This technique has been successfully applied in various endoscopic applications and shows improvement compared to conventional endoscopy in terms of visualization of cancer lesions and differentiation between neoplasia and non-neoplasia.^4^ NBI hence has been reported to improve diagnostic capabilities for application in several fields, such as diagnosis of Barrett’s esophagus,^5^ detection of colorectal lesions,^4^ and improved visualization of gastric cancer.^5^

Despite its diagnostic potential and relative simplicity in implementation, NBI has certain limitations and cannot provide sufficient information in order to replace conventional histopathology.^5^ The foremost of these are its limited resolution and restricted penetration depth, which only allows the visualization of superficial vessel networks.^6^ Furthermore, the view on the organ wall can occasionally be obscured by tissue residuals or blood.^7^ These limitations can potentially be addressed by a different imaging method—optical coherence tomography (OCT). OCT is a noninvasive imaging technique that provides cross-sectional images of tissue sites at a micrometer resolution up to a depth of ∼1.5  mm.^8^ By scanning a near-infrared beam over a two-dimensional field of view, three-dimensional (3-D) OCT volumes of the tissue of interest are acquired. Besides its well-established use in ophthalmology,^9^ OCT has also proven to be successful in endoscopic applications in cardiology,^10^ gastroenterology,^11^ and urology.^12^ In addition to the 3-D morphological information, OCT can also provide functional information about moving particles, such as blood cells, in parallel and allows the visualization of microvasculature. This label-free functional extension of OCT is referred to as optical coherence tomography angiography (OCTA). In order to perform OCTA, no hardware changes are necessary: a mere modification of the scanning protocol and a few additional steps during data postprocessing are sufficient.^13^^,^^14^ Commonly used in ophthalmic examinations of the eye or dermatology,^15^ the ability of OCTA to image vasculature of inner organs has also been demonstrated endoscopically.^6^^,^^7^^,^^16^^,^^17^ Here, OCTA has the potential to penetrate deeper compared to NBI to image vessels that are located up to ∼1.2  mm deep inside the tissue.^16^

In general, radially scanning probes are used to image tubular organs, where the scanning is performed either by a DC motor or a rotating fiber. Forward-looking variants, on the other hand, are similar to standard white light endoscopes in imaging direction, and are used to image hollow organs, e.g., the bladder.^18^ For this class of endoscopic OCT imager, the most common scanning method is a resonant fiber cantilever driven by a tubular piezoelectric actuator.^19^^,^^20^

To summarize, it can be said that OCT, especially in combination with OCTA, can provide improvements regarding the aforementioned drawbacks of NBI: (i) OCTA can visualize subsurface microvasculature, (ii) OCTA can provide volumetric 3-D datasets and therefore information about the depth location of vessels, and (iii) OCT and OCTA volume images can be merged to simultaneously display morphological as well as functional in depth information.

In this paper, we use a bimodal forward imaging probe as a tool to compare the performance of NBI and OCTA. This bimodal probe incorporates one imaging channel for full-field white light imaging (WLI) and one for 3-D OCT imaging. Consequently, the probe can be used to combine imaging technologies that complement each other. Additionally, as presented in this paper, it allows a direct comparison between NBI and OCTA by the acquisition of coregistered images. For the first demonstration of feasibility, we chose to image tissue sites that are easily accessible from the outside but lined with mucosa, as are internal organs, and selected the buccal mucosa as the tissue of interest. NBI was performed by using a ring with several green light-emitting diodes (LEDs) and OCT, OCTA and NBI images of the buccal mucosa are presented.

## 2 Methods

**Fig. 1:**
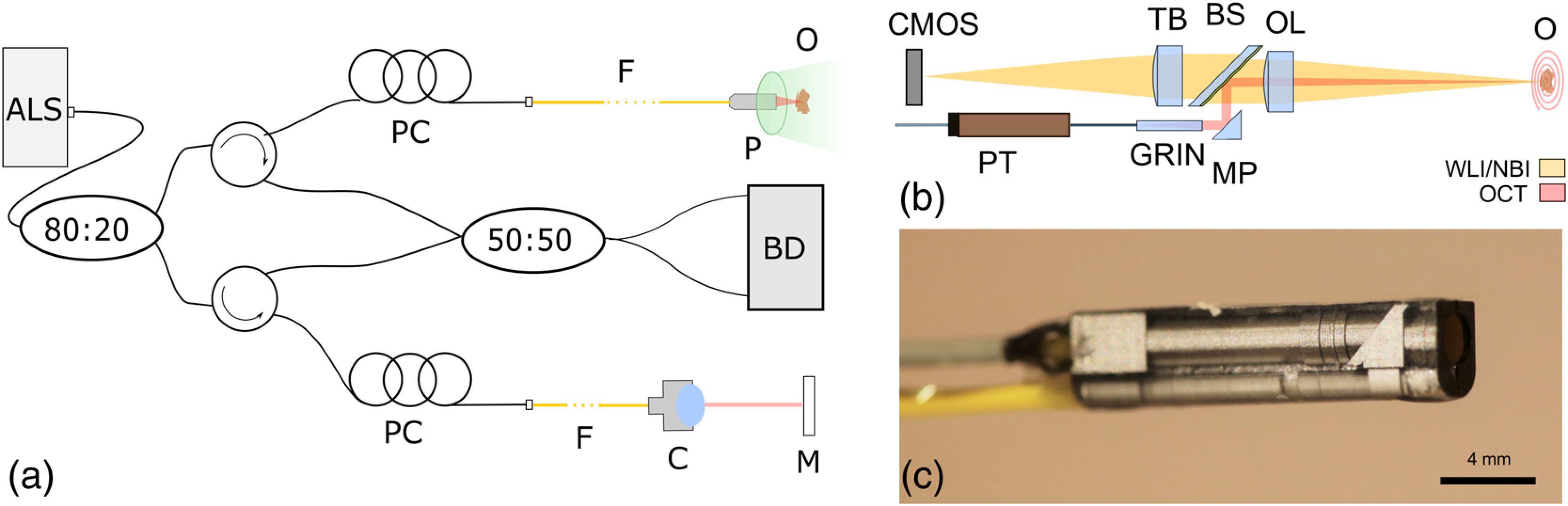
(a) Schematic of the OCT system with bimodal probe and illumination of sample with green LED light for NBI and broadband light for OCT (adapted with permission from Ref. 16, © Wiley-VCH), PC, polarization control paddles; F, fiber; C, collimator; M, mirror; P, bimodal probe; O, object; BD, balanced detection unit; ALS, akinetic light source. (b) Schematic of the optical system of the bimodal endoscope with white-light imaging channel on top and OCT channel on the bottom. PT, piezo tube; TB, tube lens; BS, dichroic beam splitter; MP, microprism; OL, objective lens. (c) Image of the coated probe.

For OCT imaging, an akinetic tunable light source from Insight Photonic Solutions was used during the experiments. This light source offers a center wavelength of 1300 nm, bandwidth of 90 nm, and was operated at a sweep repetition rate of ∼173  kHz.^21^ The output power of the source was ∼42  mW, whereas ∼14  mW power was incident on the sample using the OCT system configuration presented in Fig. 1(a). The OCT system furthermore featured an axial resolution of 12  μm. More detailed information on the imaging setup can be found elsewhere.^22^ A sensitivity of 104 dB was achieved using the OCT system without the endoscope. Introducing the endoscope and consequently additional fiber in the reference arm for compensation of path length mismatch and dispersion, a sensitivity of ∼97  dB was measured. This decrease in sensitivity is possibly attributed to increased backreflections inside the endoscope, a mismatch of the numerical aperture (NA) between the fiber of the OCT system (SMF28, NA 0.14) and the fiber of the endoscope (Thorlabs Inc. SM980G80, NA 0.18) and remaining dispersion mismatch.

The bimodal probe used for the comparison study consists of two imaging channels (see Fig. 1): a white-light imaging channel with a complementary metal-oxide-semiconductor sensor (OmniVision OV6930, 400×400  pixels, pixel size 3  μm×3  μm) at one end for acquisition of full-field images, and an OCT channel at the bottom to acquire 3-D OCT images. For OCT, scanning is performed by applying voltage to the four radial electrodes of the piezoelectric tube (PI Ceramics GmbH) that drives an attached single-mode fiber in a resonant spiral scan pattern. In terms of the optical design, the OCT beam is first collimated by a GRIN lens [GRINTECH GT-LFRL-035-025-20-NC (1550), 350  μm diameter] that is glued to the fiber tip. Consequently, the beam is deviated by a small micro prism (Optikron GmbH) to be recombined with the white light channel at a dichroic beam splitter and later focused by an objective lens. In the white light channel, a symmetrical microscope has been built by two planoconvex lenses. The optics were designed in order to provide a maximum field of view (FOV) of 1.2 mm and working distance of 6.5 mm for both beam paths. The lateral resolution of both modalities was measured using the knife-edge method and a resolution of 43.5 and 9.7  μm was determined for the OCT and WLI beam path, respectively.^23^

Both channels are encapsulated in a glass package with outer dimensions of $15\times 2.4\times 3.3\;mm^{3}$. The glass housing was manufactured by LightFab GmbH using a microstructuring process providing a precision of ±5  μm. In order to avoid any incoming stray light, the housing was covered with an opaque iridium oxide coating, as can be seen in Fig. 1(c).^23^

For the acquisition of OCTA images, the piezoelectric tube was driven at an actuation frequency of 714 Hz at maximum voltage amplitude of 42.8 V to perform a spiral scan pattern with the fiber, and the circular FOV of the image was measured to be 1.146 mm in diameter. Before each measurement, a calibration of the piezo scanner was carried out to choose the best parameters for an optimum circular scan pattern. Additionally, during this step a look-up table was stored, that was later used to reconstruct a 3-D OCT image. OCTA imaging was performed by choosing a volume rate of 0.2 Hz, but data acquisition took only place during the growing spiral scan pattern. Consequently, 1785 circular scans were obtained during one growing spiral scan in 2.5 s. This volume rate was chosen to achieve a small pitch between circular scans in order to improve the detection of signal fluctuations for OCTA.^16^ Consequently, the variance of consecutive circular scans (B-Scans) was calculated to enhance motion contrast of moving blood cells.^14^

For imaging of inner organs, the probe is indented to be used in combination with a standard endoscope by passing it through the working channel. Therefore, the light delivery system of the endoscope can at the same time be used to illuminate the sample to perform WLI or NBI with the bimodal endoscope. During our experiments, we used the bimodal probe as stand-alone component. In order to perform NBI, a ring of 16 LEDs (Lumex Standard LEDs, SSL-LX3044PGD) was built. By placing this ring around the probe head, the tissue of interest was illuminated with green diffuse light. For the acquisition of images both, the probe and the LED ring were attached to a holder mounted onto an optical table. Obviously, this configuration does not allow an acquisition of narrow-band images of inner organs. Hence, as a tissue of interest, the buccal oral mucosa was chosen, since the tissue composition in this area is similar to the tissue of inner organs. Measurements were performed by capturing NBI images while the OCTA data acquisition was running in parallel. Due to the relatively small depth of field of the WLI channel of 0.5 mm, the head of the healthy volunteer had to be placed carefully at the required distance to the distal probe head in order to obtain images in focus. Therefore, a chin rest was used to stabilize the head during the measurement procedure to meet the respective distance criteria. In addition, the chin rest also reduced subject motion during the measurement and minimized motion artifacts in acquired images.

## 3 Results and Discussion

**Fig. 2:**
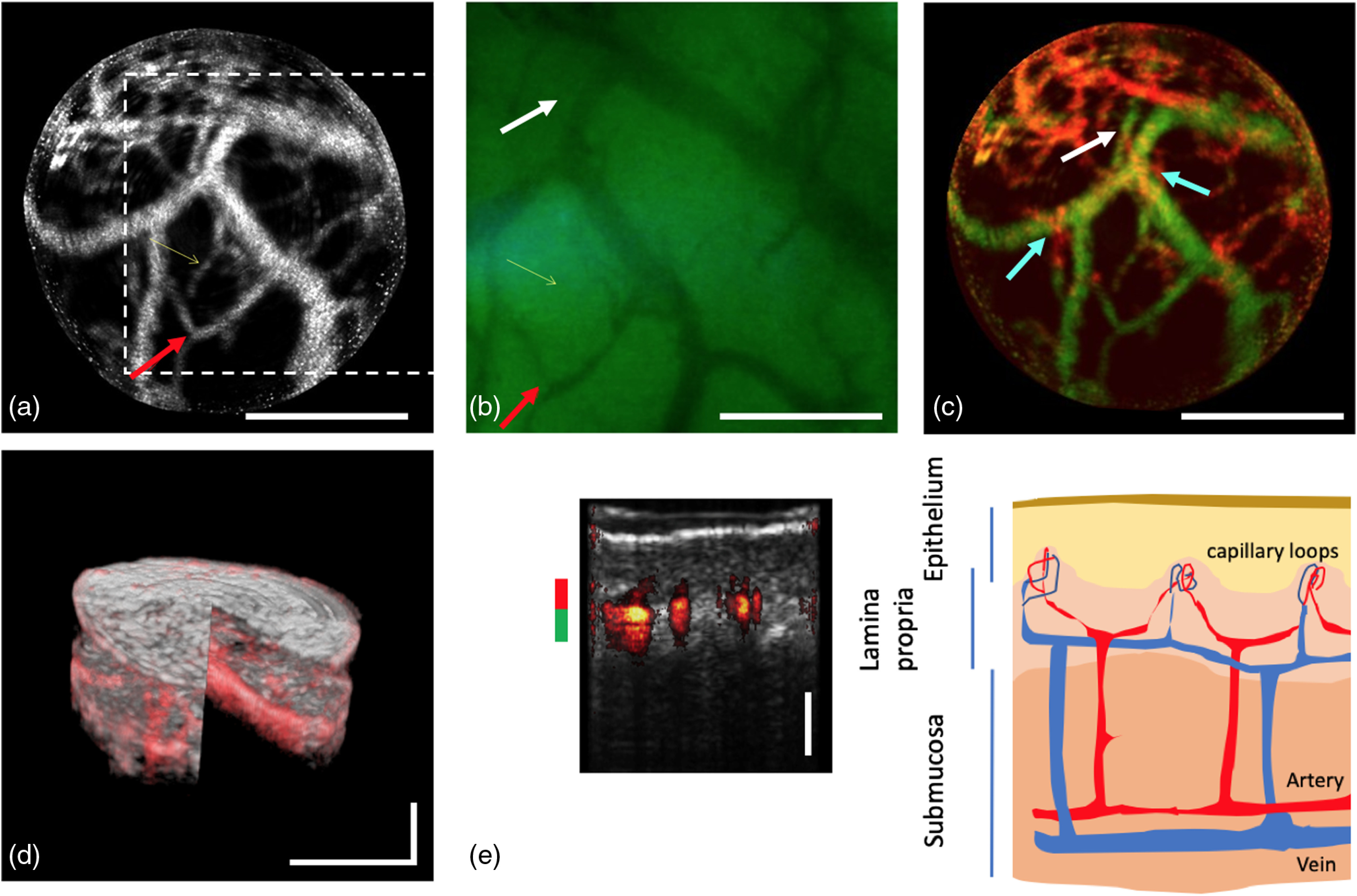
Images of the buccal mucosa obtained with the bimodal endoscope and schematic image of its structure. (a) En-face OCTA projection image at ∼200 to 444  μm depth inside the tissue with indication of NBI FOV in white dotted box. (b) Narrow-band image. (c) Depth color-coded projection image (color code in e). (d) Merged 3-D representation of OCT and OCTA data set. (e) Cross-sectional overlay tomogram (10 times averaged) and correlation to anatomy of buccal mucosa. Red arrow in (a) and (b) points out the identical vessel branch for orientation; white arrow in (b) and (c) points at a deeper located vessel that cannot be seen with NBI; cyan arrows in (c) point out vessel crossing of superficial capillaries. Scale bar: 500  μm.

Figure 2 shows different representations of images of the buccal mucosa acquired with the bimodal endoscope and for correlation a schematic drawing of the buccal mucosa’s anatomy. Informed consent from the healthy volunteer was obtained prior to the measurements, and the study was in agreement with the tenets of the Declaration of Helsinki and approved by the institutional ethics committee.

The OCTA image [Fig. 2(a)] and narrow-band image [Fig. 2(b)] allow a direct comparison between both methods. It should be noted that a slight displacement between the FOV of both imaging modalities is present [see white dotted box in Fig. 2(a)]. This was possibly caused by a design artifact in the glass housing or misalignment of the optics. Figures 2(a) and 2(c) were generated by an en-face projection of multiple slices of the OCTA data set over a depth of ∼244  μm, starting from ∼200  μm below the tissue surface until a depth of ∼444  μm. As exemplary pointed out by the red arrow, the same vessel branches are visible in the OCTA as well as narrow-band image. Figure 2(c) shows a depth color-coded projection image to visualize the different depth location of the vessels. As pointed out by the white arrow, some deeper located vessel structures can only be visualized by OCTA due to the limited penetration depth of NBI. The 3-D information is appreciated by for example observing smaller vessels and capillaries crossing above deeper larger vessels, marked by the cyan arrows in Fig. 2(c). A merged 3-D image representing OCT as well as OCTA data is presented in Fig. 2(d) correlating the morphological and function tissue information. This figure provides information about vessels and their location in depth in addition to morphological tissue information in 3-D. For further visualization, one single tomogram was chosen in Fig. 2(e) to present the structural information about the different tissue layers in addition to the depth localization of the vessels. The tissue layers can here also be correlated to the known anatomy of the nonkeratinized buccal mucosa.

Especially, the localization of larger vessel beds has proven to be significant in discrimination between different pathological conditions.^15^ However, it should be pointed out that, despite the significant higher resolution of the white-light imaging channel, OCTA enables the visualization of small microvessels. Given by the imaging principle of OCTA, even vessels whose size falls below the resolution limit can be detected.^24^ Furthermore, an endoscopic probe for OCTA has also been presented with increased lateral resolution^16^ when using a different optical design.

As an example of a pathological condition where OCT in combination with OCTA can be beneficial, an application in urology can be referenced. Although not yet standardized,^25^ several reports showed the clinical relevance of a substaging of tumor stage T1 into T1a and T1b ^26^ for an improved diagnosis of bladder cancer. T1a is associated with a tumor grow into the lamina propria but without penetrating the lamina muscularis mucosa (LMM), a thin layer consisting of vessels, nerve tissue, and muscles. In case of T1a stage, the bladder will not be removed. However, once the tumor grows into or beyond the LMM, tumor stage is referred to as T1b, a more aggressive type of tumor, and it is recommended to perform a cystectomy.^26^^,^^27^ OCT in combination with OCTA can in this situation provide a tool to distinguish between the different cancer stages by visualizing the LMM with its associated vasculature by OCTA while detecting the depth of the tumor with OCT. Several studies performed with NBI also in other organs stress the importance of visualizing the vasculature, as the structure is strongly altered in case of tumors due to the chaotic neovascular growth.^28^ However, the contrast for deeper vasculature in the submucosa is critically reduced due to the stronger scattering and absorption at the short wavelengths of NBI and can only be accessed after peeling of superficial layers.^29^ OCT and OCTA on the other hand operate at longer wavelengths and exhibit an efficient scattering suppression due to the combined confocal and coherence gating.

Still, some drawbacks of using OCTA instead of NBI should be considered: NBI is a very simple and easy to handle technique that does not require additional equipment apart from a filter. To perform OCTA on the other hand a rather complex OCT system and computational effort is required. Additionally, the FOV of current endoscopes is usually limited to a little more than 1 mm in diameter, whereas WLI endoscopes provide a much larger view on the organ. Furthermore, OCTA is very sensitive to motion. When using this probe in a more realistic setup as a handheld probe, the working distance of 6.5 mm and the relatively slow volume rate of would certainly result in significant motion artifacts that would strongly affect the quality of OCT and OCTA. Alternatively, MHz endoscopic imaging has been shown recently,^20^ with high volume rates, that could easily avoid the deleterious effect of motion on OCT and OCTA. On the other hand, long working distances can be avoided by designing the optics such that the probe stays in contact with the tissue as has been demonstrated in Ref. 16.

## 4 Conclusion

In conclusion, we were able to perform a preliminary study for comparison of endoscopic OCTA and NBI. Therefore, a bimodal endoscope that allowed simultaneous acquisition of OCT/OCTA and narrow-band images was used to obtain coregistered images of the microvasculature of the buccal mucosa. Furthermore, a cross-sectional overlay image was correlated with the known structure of the buccal mucosa. The obtained results demonstrate that endoscopic OCT and OCTA are able to visualize tissue structure and function in 3-D with quantitative information about the depth location of specific vascular structures that might enhance current diagnostic capabilities of video endoscopy. The information on the invasiveness of malignant tissue or depth of suspicious lesions is expected to improve the early detection and stage assessment of malignant diseases of inner organs. This has an important impact on further treatment decisions. In order to promote improvements regarding the diagnostic capabilities of OCT, further validation in future studies is needed by imaging specific inner organs with deeper located microvasculature.
